# Experimental Comparison of Fiducial Markers Used in Proton Therapy: Study of Different Imaging Modalities and Proton Fluence Perturbations Measured With CMOS Pixel Sensors

**DOI:** 10.3389/fonc.2022.830080

**Published:** 2022-03-25

**Authors:** Claire-Anne Reidel, Felix Horst, Christoph Schuy, Oliver Jäkel, Swantje Ecker, Katrin Henkner, Stephan Brons, Marco Durante, Uli Weber

**Affiliations:** ^1^ Biophysics Department, GSI Helmholtzzentrum für Schwerionenforschung GmbH, Darmstadt, Germany; ^2^ Division of Medical Physics in Radiation Oncology, German Cancer Research Center (DKFZ), Heidelberg, Germany; ^3^ Heidelberg Ion Beam Therapy Center (HIT), Heidelberg, Germany; ^4^ Institut für Physik Kondensierter Materie, Technische Universität Darmstadt, Darmstadt, Germany

**Keywords:** fiducial marker, dose perturbation, proton therapy, CMOS pixel sensor, image guidance, streak artifacts, Monte Carlo simulation

## Abstract

Fiducial markers are used for image guidance to verify the correct positioning of the target for the case of tumors that can suffer interfractional motion during proton therapy. The markers should be visible on daily imaging, but at the same time, they should produce minimal streak artifacts in the CT scans for treatment planning and induce only slight dose perturbations during particle therapy. In this work, these three criteria were experimentally investigated at the Heidelberg Ion Beam Therapy Center. Several small fiducial markers with different geometries and materials (gold, platinum, and carbon-coated ZrO_2_) were evaluated. The streak artifacts on treatment planning CT were measured with and without iMAR correction, showing significantly smaller artifacts from markers lighter than 6 mg and a clear improvement with iMAR correction. Daily imaging as X-ray projections and in-room mobile CT were also performed. Markers heavier than 6 mg showed a better contrast in the X-ray projections, whereas on the images from the in-room mobile CT, all markers were clearly visible. In the other part of this work, fluence perturbations of proton beams were measured for the same markers by using a tracker system of several high spatial resolution CMOS pixel sensors. The measurements were performed for single-energy beams, as well as for a spread-out Bragg peak. Three-dimensional fluence distributions were computed after reconstructing all particle trajectories. These measurements clearly showed that the ZrO_2_ markers and the low-mass gold/platinum markers (0.35mm diameter) induce perturbations being 2–3 times lower than the heavier gold or platinum markers of 0.5mm diameter. Monte Carlo simulations, using the FLUKA code, were used to compute dose distributions and showed good agreement with the experimental data after adjusting the phase space of the simulated proton beam compared to the experimental beam.

## 1 Introduction

Over the last years, innovative techniques for particle therapy were developed in order to deliver a more conformal dose to the tumor and better spare healthy tissues. A mispositioning of the patient can lead to severe under- and overdosage, especially for proton and ion beams where high doses are delivered at the end of their range (1, 2).

During radiation therapy, the positioning of the patient is ensured by daily imaging, usually performed by X-ray projections, cone beam computed tomography (CBCT), or in-room mobile CT. In most cases, a patient is aligned to the absolute coordinate system of the treatment room by matching its bony structure, visible on the daily image, to the one reconstructed from the treatment planning CT. In the case of interfractional motion due to anatomical changes, e.g., prostate cancer, the tumor can move in the range 0–2 cm due to the filling of the bladder and rectum (3–5). Therefore, fiducial markers are implanted inside or nearby the tumor before the treatment and are used for image guidance during radiation therapy (6, 7) since the alignment with the bony structure of the patient is not reliable for tumor position in some regions. Their position on the daily image is compared to the one from the treatment planning CT, and the consistency of the tumor position is assessed to decide if the treatment can be performed or if corrections are necessary.

Several criteria are to be considered for the fiducial markers. Three important ones were evaluated in this study: low streak artifacts on the treatment planning CT, good visibility on the daily images, and low perturbation of the dose distribution during particle therapy. For a good visibility on the daily image, the markers are generally composed of high density and high atomic number materials such as gold or platinum, but also fiducial markers with lower-density material such as zirconium dioxide (ZrO_2_) were considered (8–10). However, due to their high atomic number and density, metallic markers induce streak artifacts on the treatment planning CT and may cause errors in the dose calculation during treatment planning (11). Several studies have been performed to evaluate the visibility of different markers and the streak artifacts that they produce, showing that high-density markers are necessary in order to be visible on X-ray projections (8, 9). In addition to streak artifacts, high-density fiducial markers induce dose perturbations and additional range uncertainties during particle therapy, due to inhomogeneous scattering through high-density gradient edges and their different stopping power relative to water, respectively. The multiple Coulomb scattering depends on the projectile species and energy, as well as the material dimensions and composition, and can be estimated by the Highland formula (12, 13). In previous studies, the severeness of this effect was evaluated for different markers by Monte Carlo simulations (14–16) and/or by measurements with radiochromic films (17–19). Another experimental study, conducted by our group, quantified the fluence perturbation of carbon ions for different fiducial markers with an advanced measurement technique, using CMOS pixel sensors (20). These studies showed that the strength of the perturbation mostly depends on the size and density of the fiducial marker. The ZrO_2_ marker showed less perturbation than did gold markers of comparable size. On the other hand, this marker was less visible on the daily X-ray projection imaging. The present work intends to investigate these effects and to discuss the trade-off between the visibility versus the dose perturbations during proton therapy, which is more frequently used than carbon ion therapy. Moreover, perturbations are larger for protons than carbon ions due to the stronger multiple Coulomb scattering, which gives an additional motivation for this work.

In the present work, a comprehensive study, including imaging, experimental measurements for proton beams, and Monte Carlo simulations, was performed for various fiducial markers made of different materials and geometries. In a first part, an imaging study was carried out to evaluate the streak artifacts on the treatment planning CT, and the visibility on X-ray projections and on the images taken with an in-room mobile CT. In a second part, fluence perturbations of proton beams due to fiducial markers were measured with CMOS pixel sensors, where the maximum perturbation and its position along the beam axis were quantified. Both experimental parts were conducted at the Heidelberg Ion Beam Therapy Center (HIT) (21). For the fluence perturbation measurements, a set of high spatial resolution Mimosa-28 pixel sensors was used (22), as in our previous work (20). In a last part, Monte Carlo simulations, performed with the FLUKA code (23–25), were compared against the experimental results, and dose perturbations in a spread-out Bragg peak (SOBP) were computed for the same markers and proton beams as in the experiment.

## 2 Materials and Methods

### 2.1 Fiducial Markers

The experimental study was performed for 8 fiducial markers clinically in use, with their properties listed in **Table 1**. The folded Gold Anchor markers were evaluated during the imaging study only, while the linear Gold Anchor was used during the proton beam experiment because of its more defined geometry. All the other listed markers were used in both experiments. In the case of the Visicoil markers, the length and diameter were given by the manufacturer and each marker was weighted. The inner diameter, referred in **Table 1**, was calculated and adjusted compared to the mass of the marker.

**Table 1:** Properties of all fiducial markers used in this study for the imaging and proton beam experiments.

Marker	Name	Manufacturer	Material	Shape	Length	Diameter	Mass
number					(mm)	(mm)	(mg)
1	Visicoil	RadioMed	Gold	Coiled	5	0.35*^a^*	3.8
2	Visicoil	RadioMed	Gold	Coiled	5	0.5*^b^*	10.8
3	Visicoil	RadioMed	Platinum	Coiled	5	0.35*^c^*	3.6
4	Visicoil	RadioMed	Platinum	Coiled	5	0.5*^d^*	12.6
	Gold Anchor	Naslund	Gold	Linear	15	0.28	12.3
		Medical AB					
6	Gold Anchor	Naslund	Gold	Folded	10	0.28	7.3
		Medical AB					
7	Gold Anchor	Naslund	Gold	Folded	20	0.28	14.2
		Medical AB					
8	Acculoc	Carbon Medical	ZrO_2_	Bone	3	1	5.5
	Carbon marker	Technologies	(carbon-coated)				

Inner diameter calculated according to the mass (mm).

^a^0.27, ^b^0.33, ^c^0.28, ^d^0.32.

### 2.2 Imaging Study

**Figure 1:**
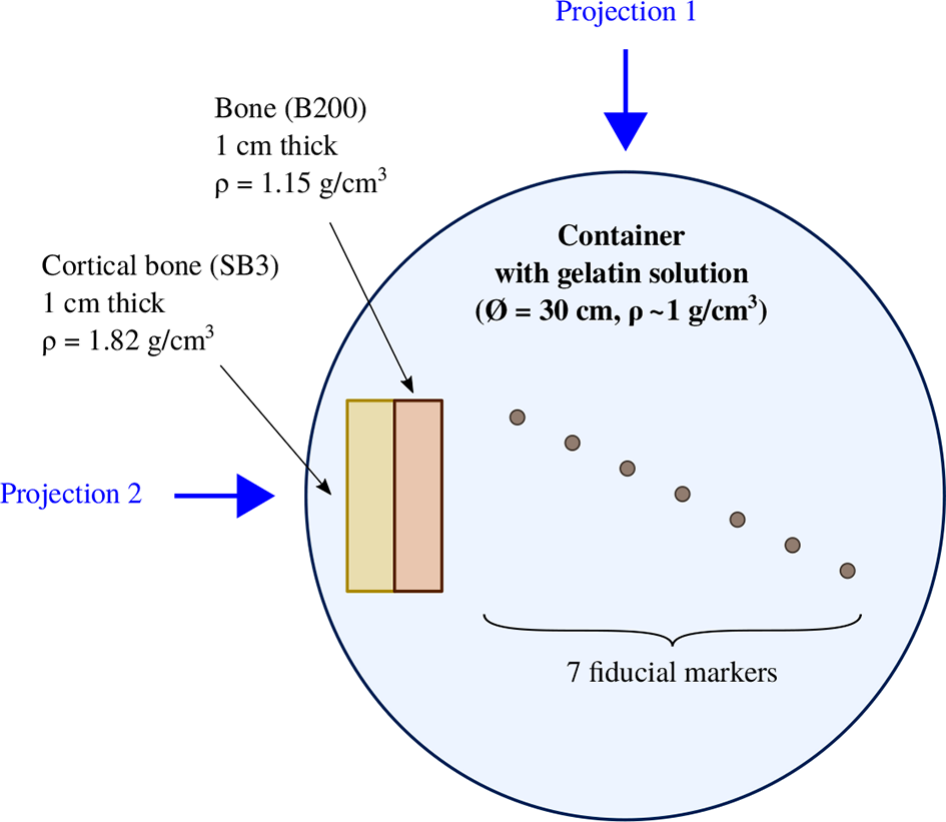
Phantom used for the imaging study where 7 fiducial markers were inserted inside a gelatin solution. Two bone-like slabs were placed on one side of the phantom.

The imaging was performed for 7 fiducial markers [all markers listed in **Table 1**, except the linear Gold Anchor (5)]. This study was conducted at HIT, using the treatment planning CT, the recently installed in-room mobile CT, and the standard X-ray projection method for daily imaging (see specifications in the sections below). The fiducial markers were inserted in a container of around 30 cm diameter, filled with a homogeneous gelatin solution. To obtain a more realistic case, two bone-like slabs of different density, manufactured by GAMMEX, were placed at one side of the phantom. The X-ray images were performed using two projections, one traversing the bone materials that shadowed the markers, and a second offset by 90° where the bone material does not shadow the markers. Therefore, it was possible to compare the X-ray projections with and without bone slabs in front of the markers. A schematic of the phantom used for the imaging study is sketched in **Figure 1**.

#### 2.2.1 Streak Artifacts on Treatment Planning CT

The CT scans were acquired by a SOMATOM Confidence^®^ scanner (Siemens, Erlangen, Germany), using the standard protocol for head planning CT at HIT, with 120 kVP, 255 mAs, and 500 mm field of view. The transversal pixel resolution was 0.977 mm, and the scans were performed with 0.5 and 1mm slice thicknesses, with and without applying an iterative metal artifact reduction (iMAR) correction in the reconstruction. The images were later analyzed with the software ImageJ (26). A square of 2mm length was drawn at the marker position where the Hounsfield value was maximum. Square-shaped rings with a thickness of one pixel (~1 mm) and with an inner length as the one of the previous ring were then drawn around the marker (see **Figure 3C**). The streak artifacts, defined as the maximum and minimum values inside the different square-shaped rings, were computed as a function of the distance from the marker position.

#### 2.2.2 Visibility on X-Ray Projections and In-Room Mobile CT Images

A qualitative comparison of the visibility from the different fiducial markers on X-ray projections was performed with an AXIOM Artis (Siemens) robotic arm, installed in the treatment room at HIT (8). This on-board imaging device is used for patient position verification prior to the treatment. In this study, imaging was performed with and without collimation, and with and without the bone slabs in front of the markers. The settings of the X-ray machine for the different acquisitions are listed in **Table 2**.

**Table 2:** Settings of the X-ray machine for the different acquisitions of fiducial marker images at HIT.

In-line bone	kVP	Current time product (mAs)	Exposure time (ms)	Collimation
No	69	172	59	No
No	69	254	94	Yes
Yes	69	176	61	No
Yes	69	279	105	Yes

The images analysis was then performed with the software ImageJ (26). A rectangular window of 3mm height and 6mm width was drawn perpendicularly to each marker placed in vertical position. The profile was integrated within this window, and the maximum value *I_max_* was extracted from the profile. In order to evaluate the background, a window of identical size as the previous one was drawn close to the marker position. In this work, the background *I_b_* was computed as the mean value of the integrated window, while the pixel noise was defined as the standard deviation σ. The errors on *I_max_* and *I_b_* were considered the same, and the contrast *C* was then computed as


(1) $$C=|I_{\max}-I_{b}|\pm \;\sigma \;\sqrt{2/N},$$

where *N* is the sample number in the window area.

The in-room mobile CT images were obtained with an AIRO (Mobius Imaging, LLC), which is the in-room mobile CT installed in one of the treatment rooms at HIT. The scans were performed with 120 kVP, 80 mAs, and 493 mm field of view. The transversal pixel resolution was 0.963 mm for a slice thickness of 1 mm.

### 2.3 Fluence Perturbation Measurements

#### 2.3.1 Mimosa-28 Pixel Sensor and Software Analysis

The Mimosa-28 (Minimum Ionizing MOS Active pixel sensor) detector, based on CMOS technology, is a high spatial resolution pixel sensor (22). The sensor has an active area of ~ 2 × 2 cm^2^ and is composed of 928 rows × 960 columns with squared pixels of 20.7μm length. The total thickness of the sensor is 50 μm, with an epitaxial layer of 14 μm. Each pixel delivers a binary output after discrimination of the signal, and the sensor has a readout time of 186.5 μs (~5 kHz frame rate).

When a particle passes through the sensor, charges produced by ionization are collected by a certain number of pixels in the sensor. The analysis software Qapivi (27), based on the Root (28) and Geant4 ( 29) libraries, reconstructs the groups of fired pixels, referred to as clusters. The cluster position is defined as the center of mass of the group of fired pixels, and a straight line (called track) matching the clusters in the different sensors is reconstructed. The tracking procedure was performed with the implemented algorithm based on multiple Coulomb scattering. The resolution of a single track is better than 10 μm, and the performance of the algorithms is described in the study from (30). In order to reach a high track resolution, it is necessary to align the sensors using a dedicated no-target run to compensate mechanical mispositioning of the sensors *via* a software alignment procedure (31).

#### 2.3.2 Experimental Setup

**Figure 2:**
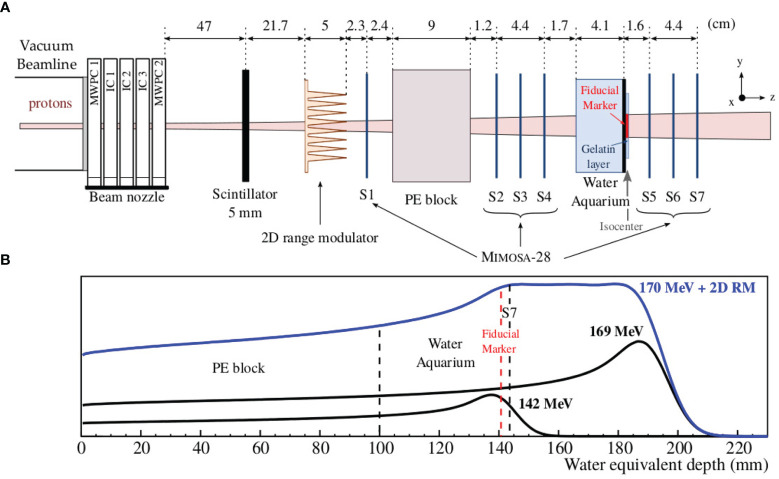
**(A)** Experimental setup for fluence perturbation of proton beams due to fiducial markers, measured with 7 Mimosa-28 pixel sensors. **(B)** The depth–dose profiles in water equivalent are shown for 142 and 169MeV protons, as well as for the 170-MeV proton beams modulated by a 2D RM. The depth–dose profiles were laterally integrated and obtained with the Monte Carlo code FLUKA (23-25). In this panel, the PE block, the water aquarium, and the positions of the fiducial marker and of the last sensor S7 are also indicated.

The fluence perturbation measurements of proton beams due to fiducial markers were conducted in the experimental room at HIT, placing a tracker system of 7 Mimosa-28 pixel sensors along the beam axis. The experimental setup was similar to the one used in our previous work (20). A water aquarium of 4cm length, representing the tumor volume, was positioned in between two sets of three sensors. To improve their handling, the markers were glued to a thin polymethyl methacrylate (PMMA) plate of 1mm thickness and 1.18g/cm^3^ density. The markers were positioned along the vertical axis perpendicular to the beam at the isocenter of the experimental room. The PMMA plate, including the marker, was glued behind the water aquarium. In our last study with carbon ion beams, the marker was immersed in the water aquarium. However, the maximum perturbation of proton beams was expected to be closer to the marker; therefore, their position was adjusted in order to place the tracker system closer to the marker. The markers were surrounded by a thin layer of gelatin solution to have a more realistic setup where the edges of the marker were surrounded by tissue-like material. In addition, a polyethylene (PE) block of 9cm length was placed in front of the first set of three sensors to simulate the healthy tissues of a patient. Another sensor was positioned in front of the PE block in order to monitor the stability of the beam profile between different measurements, and a 5mm plastic scintillator (BC-400) monitored the beam intensity by counting the incoming particles. A range modulator (2D RM) (32) was placed in front of the first Mimosa-28 sensor. The modulator used in the present work was optimized for a proton SOBP of 5 cm and is composed of 13 × 13 pins within an area of around 4 × 4 cm^2^. The pin length is 5 cm, and the modulator is composed of Rigur, which is a polypropylene-like material for 3D printing. The experimental setup is depicted in **Figure 2A**.

The beam time campaign was divided in several parts. First, the runs were performed only with the 7 Mimosa-28 sensors and the plastic scintillator in order to properly align the sensors and tune the initial beam parameters for the Monte Carlo simulations. Second, the measurements were performed with the PE block and the water aquarium to obtain a reference measurement without marker. In a next step, the fiducial markers were placed behind the water aquarium, as explained above. These measurements were performed with two single-energy proton beams (142.10 and 169.02 MeV), which were chosen to have a range difference of 5 cm. In a last step, the 2D RM was positioned in front of the first sensor and the measurements were performed with the PE block and the water aquarium, with and without fiducial marker. For this set of measurements, a single proton energy of 170.05 MeV together with the 2D RM was used to produce a 5cm SOBP. The primary energy was chosen slightly higher than 169.02 MeV because of the additional base plate of the 2D RM. The primary beam energies, their full width half maximum (FWHM), and their range in water are listed in **Table 3**. The energies and FWHM at isocenter position were assumed as the nominal values from HIT (ensured by the regular QA), while the ranges were calculated with LISE++ (33). In **Figure 2B**, the depth–dose profiles in water equivalent are shown for 142 and 169MeV protons, as well as the SOBP produced by the 2D RM for a primary beam of 170MeV protons. The PE block, the water aquarium, and the positions of the fiducial marker and of the last sensor S7 are also indicated. The total range of the different proton beams used in the experiment was chosen to have enough energy to pass through the PE block, the water aquarium, and the sensors placed behind the water aquarium.

**Table 3:** Primary beam energy, FWHM at the isocenter, and range in water (calculated with LISE++) of the proton beams used for the measurements.

Energy (MeV)	FWHM (mm)	Range in water (mm)
142.10	11.7	143.6
169.02	10.0	194.3
170.05	10.0	196.4

#### 2.3.3 Beam Profile Analysis

The beam profiles, measured with the Mimosa-28 pixel sensors, were used to validate the Monte Carlo simulations. The beam profiles were extracted from the cluster maps, defined by the position of all clusters in *x* and *y*, for all 7 CMOS pixel sensors placed at different positions along the *z*-axis (see **Figure 2A** for the coordinate system). The beam profiles in *x* and *y* were obtained after integrating (averaging) the cluster maps over the perpendicular directions *y* and *x*, respectively. The profiles were used to adjust the initial beam parameters of the Monte Carlo simulations for 142.10 and 170.05 MeV. In a next step, the beam profiles were computed with the fiducial markers. For this, the profiles were obtained after integrating the cluster maps over a given window along the *y*-axis that was chosen according to the marker length.

#### 2.3.4 Fluence Distribution Analysis

This study aims at determining the maximum perturbation and its position along the beam axis with high spatial resolution. For this, a three-dimensional (3D) fluence distribution was computed after reconstructing the trajectory of each particle crossing the tracker system. This distribution was computed from all tracks, which are defined by 3D vectors, reconstructed with the tracker placed behind the aquarium (sensors S5–S7 in **Figure 2A**), and extrapolated to the fiducial marker position. Voxels of 20 × 20 × 200 m^3^ were defined, and the fluence in each voxel was determined as the sum of all the tracks passing through this voxel. Therefore, the 3D fluence distribution scores the total number of intersections between the reconstructed tracks and the voxels. The 2D fluence distributions (referred to as fluence maps), extracted from the 3D fluence distributions, are presented in this study to quantify the propagation of the perturbation in the (x,z) plane. The fluence map was computed by integrating the tracks over a given window along the *y*-axis that was chosen according to the marker length. From the integrated fluence map, the perturbation at any position along the beam axis can be assessed. The fluence maps with and without marker were reconstructed, and the beam profiles were extracted at the position along the beam where the perturbation was maximum. The maximum fluence perturbation was then quantified by comparing the beam profiles (*x,y*) with and without marker. The fluence maps were computed for the case of single-energy beams and for the modulated beam by the 2D RM. It is important to note that during the experiment the beam moved due to unintended drift effects of the ion optics, which made it difficult to use the reference measurement, in particular for the 2D RM case. Therefore, the beam profile from the reference measurement could not be used to quantify the perturbation. For this case, the beam profile of the corresponding maximum perturbation was computed, and the reference was defined as the fit of the profile without taking into account the perturbation. Since the beam was slightly shifted and tilted compared to the tracker system, the beam profiles obtained after the tracking were not perfectly Gaussian-like. Therefore, they were fitted by convoluting three Gaussian functions. This method was verified and validated on the beam profiles with a single energy, where the reference measurement could be used properly.

### 2.4 Monte Carlo Simulations

The Monte Carlo simulations were performed with the FLUKA2020 code version 0.10 (23–25). The default PRECISIO settings were used. In FLUKA, single Coulomb scattering events are condensed in a multiple scattering algorithm. Fluence and dose profiles were calculated using the USRBIN scorer.

#### 2.4.1 Setup Geometry

The setup geometry of the FLUKA simulations reproduced the one used during the experiment. For this, several layers of different thicknesses and materials were placed along the beam axis. The beam nozzle, which is composed of several detectors and air gaps, was simulated by a water volume of 320mm length and 0.0115 g/cm^3^. The Mimosa-28 sensors were simulated by a silicon volume of 50μm thickness, and the scoring volume was defined as the sensitive layer of the sensor of 14-μm thickness. The sensor is surrounded by a printed circuit board (PCB) of 1.7mm thickness. The PCB of the sensor was also implemented in the simulation since low-energy particles can be stopped in this material layer. The primary energy of the proton beams was chosen as the ones used during the experimental campaign (see **Table 3**). The simulations were also performed for an initial beam shift (and tilt), which means that the beam position and divergence in front of the exit window were set as the ones obtained from the CMOS measurements. The fiducial markers were designed as tubes for the 4 Visicoil markers and as cylinders for the Gold Anchor and the ZrO_2_ markers. The approximations of the markers as cylinder/tube neglect the helical structure of the Visicoil and the bone shape of the ZrO_2_ markers. Considering the size of these fine structures compared to the resolution of the scoring grid, those assumptions are believed to be reasonable. The dimensions used for the Monte Carlo simulations are indicated in **Table 1**.

#### 2.4.2 Benchmarking

To benchmark the Monte Carlo code FLUKA against the Mimosa-28 pixel sensor measurements, the optical beam parameters (FWHM and divergence) were tuned to reproduce the measured lateral beam spread along the beam axis. The transport code Scattman (1, 34) was used to extract the ion optical parameters (phase space), in particular the beam width and divergence in front of the vacuum exit window. The parameters were obtained based on the measured beam profiles and were used as initial parameters for the Monte Carlo simulations. The beam profiles from the Monte Carlo simulations were computed in the scoring volume, which represents the CMOS sensors, at different positions along the beam axis. First, the simulated beam profiles were compared to the measured ones on one hand without any target and on the other hand with the PE block and the water aquarium. Second, the simulations were performed with the PE block, the water aquarium, and the fiducial marker. For these simulations, the PCB of the sensor and the initial beam tilt and shift were also implemented. In addition, the primary beam energy of the Monte Carlo simulations was verified to the one calculated from the energy loss estimation in the sensors (35). To compare the perturbation from the fiducial markers, the 2D beam profiles were extracted at each sensor position for the simulated and experimental results. The beam profiles were then computed after integration over a given window along the *y*-axis that was chosen according to the marker length. The perturbations were quantified by comparing the beam profiles with and without fiducial markers.

#### 2.4.3 Dose Distribution

The dose distributions were computed with a simple setup composed of a water phantom and the fiducial markers implanted at 15cm depth. A 5cm length SOBP from 14 to 19 cm (lateral extensions 5 × 5 cm^2^) was simulated with several proton pristine Bragg peaks. The lowest and highest energies were set to 142 and 169 MeV as the ones used during the experiment, respectively. The dose distributions in the water phantom were scored as 2D maps to quantify the cold spots. The resolution of the 2D maps was 0.02 mm in *x* and 0.4 mm in *z*, after integrating over the length of the marker in *y*. A reference simulation without marker was also performed to quantify the cold spot. The maximum cold spot and its position along the beam axis could then be evaluated.

## 3 Results

### 3.1 Imaging Study

#### 3.1.1 Treatment Planning CT Streak Artifacts

**Figure 3:**
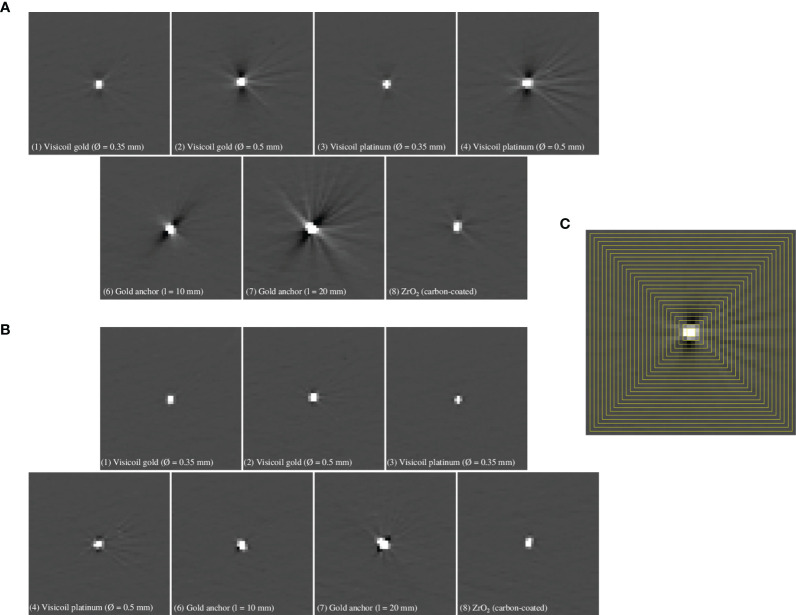
Reconstructed images for conventional treatment planning CT **(A)** and with iMAR correction **(B)** of 0.5mm slice thickness for 7 different fiducial markers (**Table 1**). **(C)** Square-shaped rings (yellow lines) used to determine the minimum and maximum values as a function of the distance from the marker, as explained in *Section 2.2.1*.

The images from the treatment planning CT of 0.5mm slice thickness with and without iMAR correction are presented in **Figure 3** for 7 different markers (see **Table 1**). It is important to note that the markers are smaller than one voxel in the reconstruction. Therefore, the maximum HU values are determined by the marker density and partial volume effects.

**Figure 4:**
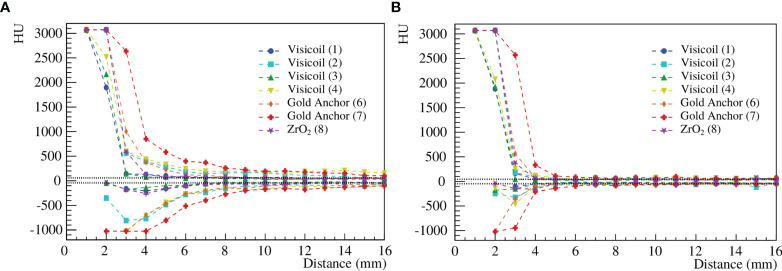
Maximum and minimum HU as a function of the distance to the fiducial marker for the conventional treatment planning CT **(A)** and with iMAR correction **(B)** for 7 fiducial markers (**Table 1**).

The maximum and minimum gray levels of the recorded images with the treatment planning CT were computed for 7 fiducial markers, as described in Section 2.2.1. In **Figure 4**, the minimum and maximum values in Hounsfield units (HU) are shown as a function of the distance to the fiducial marker for the conventional planning CT and the one with iMAR correction.

For all fiducial markers, the maximum value reaches 3060 in the marker area, while for the Gold Anchor also -1024 is reached, even if the markers fill only a partial volume of the reconstructed voxels. Before treatment planning, these saturated voxels need to be overwritten by a realistic value. In **Table 4**, the distance from the fiducial marker position, at which the maximum and minimum values of the streak artifacts become lower than 3% of the background level, is summarized for the 7 fiducial markers for the conventional treatment planning CT and the one with iMAR correction. The folded Gold Anchor of 20mm length (7), which is also the heaviest marker, shows the strongest streak artifacts. The Visicoil markers (1) and (3) of 0.35mm diameter (gold and platinum) and the ZrO_2_ marker (8), which are the lighter studied markers, are the ones producing less intense streak artifacts. The Viscoil markers (2) and (4) of 0.5mm diameter and the Gold Anchor of 10mm length (6) show similar values. **Figure 4B** shows that streak artifacts are significantly reduced when using an iMAR correction. The HU drops down to the background level around the marker, while for the conventional treatment planning CT, the artifacts propagate at further distances from the marker. Therefore, the application of an iMAR correction could significantly reduce the errors in dose calculation due to fiducial markers and should be investigated further in the future.

**Table 4:** Distance from the fiducial marker position, at which the streak artifacts become lower than 3% of the background level for 7 fiducial markers (**Table 1**), for the conventional treatment planning CT and the one with iMAR correction.

Marker number	1	2	3	4	6	7	8
CT distance artifacts	7	16	7	19	11	18	7
(mm)								
CT distance artifacts	4	5	4	5	5	6	5
iMAR (mm)								

#### 3.1.2 X-Ray Visibility

**Figure 5:**
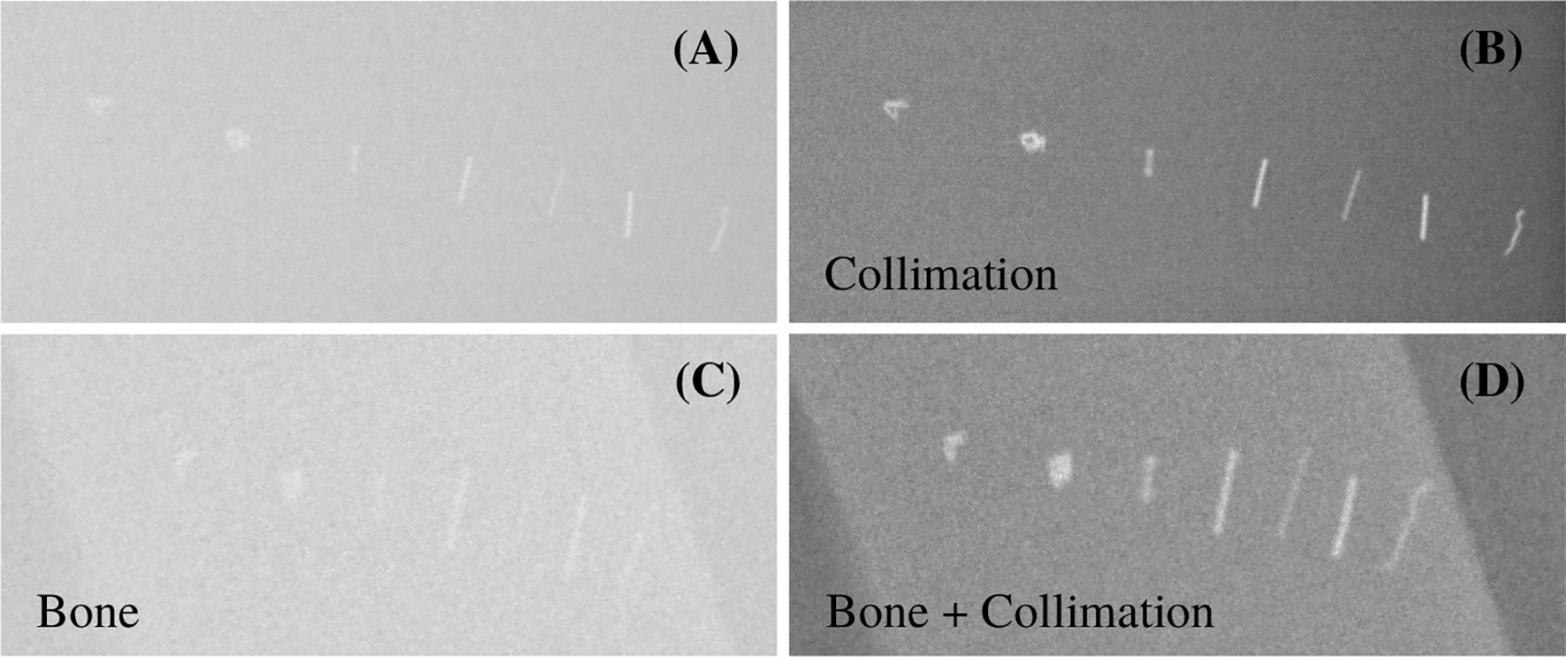
X-ray images of 7 different fiducial markers for the different settings described in **Table 2**. From left to right: Gold Anchor (6), Gold Anchor (7), ZrO_2_ (8), Viscoil (2), Visicoil (3), Visicoil (4), and Visicoil (1), listed in **Table 1**. **(A, B)** show the images without and with collimation. **(C, D)** show the images with the bone slabs in front of the markers without and with collimation, respectively.

**Figure 6:**
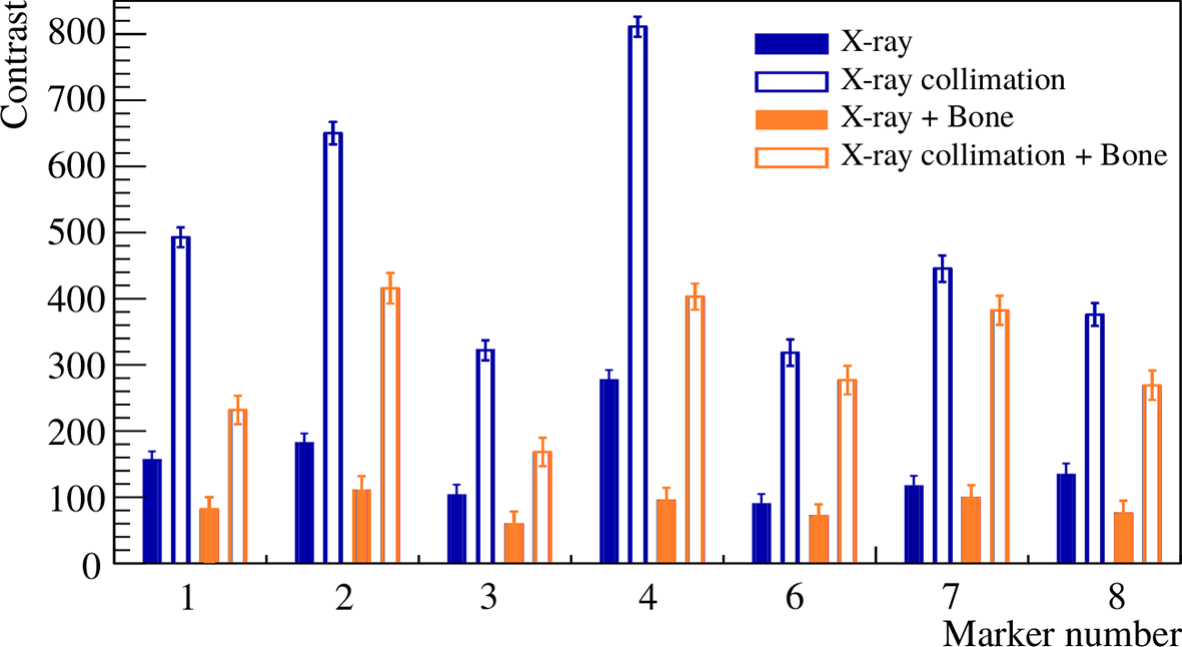
Contrast of different fiducial markers (listed in **Table 1**) from the X-ray imaging study for the different settings of **Table 2**.

The images acquired during the X-ray measurements are shown in **Figure 5** for the different scenarios with and without collimation, as well as with and without bone slabs placed in front of the markers. The contrast of the different fiducial markers was computed as described in Section 2.2.2. In **Figure 6**, the contrast of 7 fiducial markers is shown for the X-ray imaging with the 4 different scenarios.

**Figure 7:**
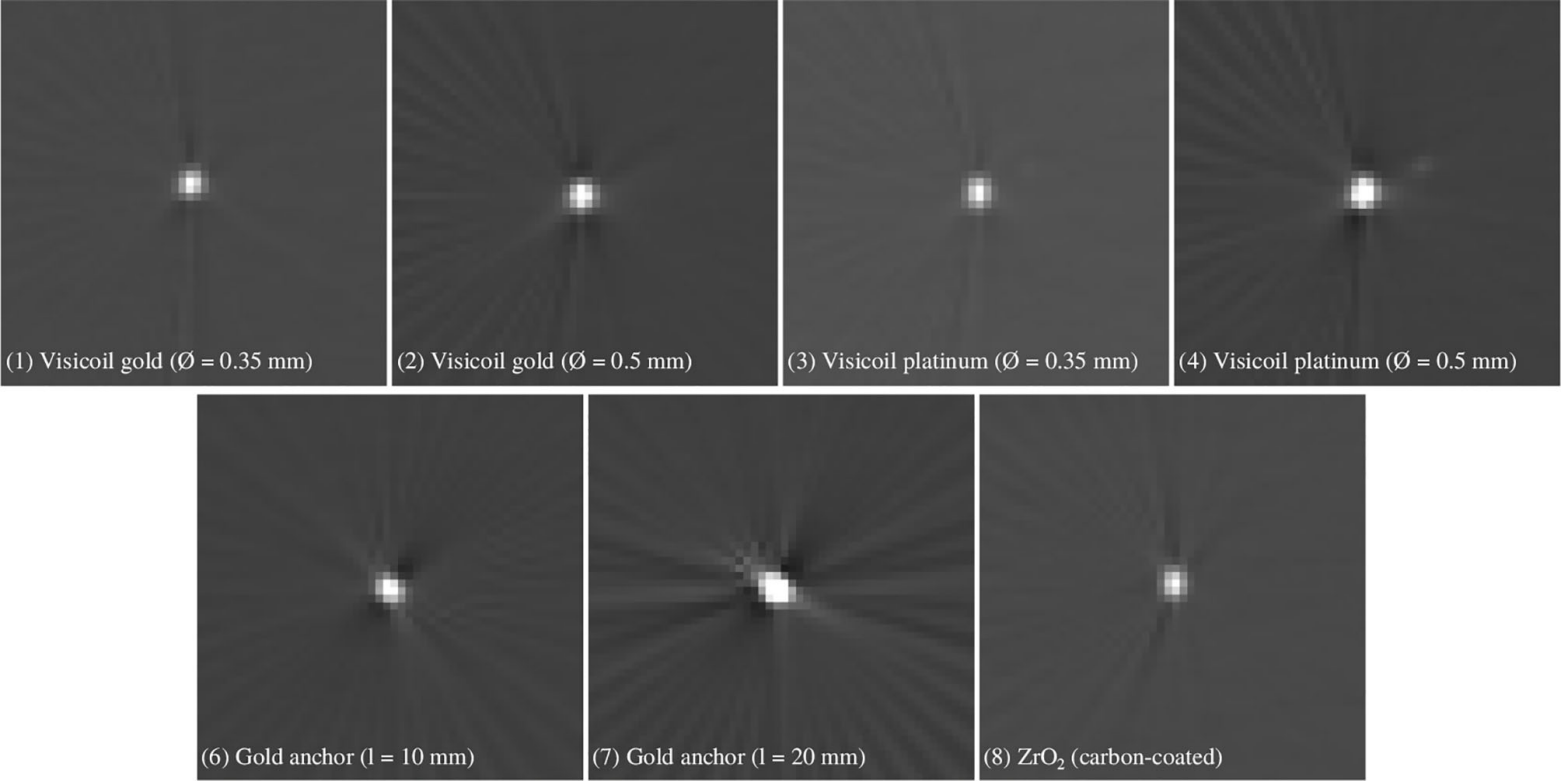
In-room mobile CT images of 7 different fiducial markers listed in **Table 1**.

**Figure 6** shows that the contrast is significantly improved when a collimation is applied during the imaging, which is the common practice in order to reduce the patient exposure. In a more realistic case, where a bone slab was placed in front, the contrast decreases. The heavier markers, with high-density materials and a mass larger than 10 mg, such as the Gold Anchor (7) and the two Visicoil markers of 0.5mm diameter (2) and (4), show a contrast of around 400. For the Gold Anchor (6) and the ZrO_2_ (8) of mass between 5 and 10 mg, the contrast is around 300. For the smaller Visicoil markers (1) and (3) of mass lower than 5 mg, the contrast is around 200. However, all markers are visible on the images. The images from the in-room mobile CT study are shown in **Figure 7** for 7 fiducial markers. All markers are clearly visible with this imaging method. Streak artifacts are also present, but not particularly important when the images are not used for dose calculation.

### 3.2 Fluence Perturbation: CMOS Measurements

Fluence perturbations due to the fiducial markers are induced by multiple Coulomb scattering, which means that the perturbation varies along the beam axis. In addition, for low-energy beams, fiducial markers can also create considerable range shifts due to energy losses in the markers. In this section, the results of the maximum perturbation due to the fiducial markers were computed from the fluence map of all reconstructed tracks, as explained in *Section 2.3.4*.

#### 3.2.1 Single-Energy Proton Beam

**Figure 8:**
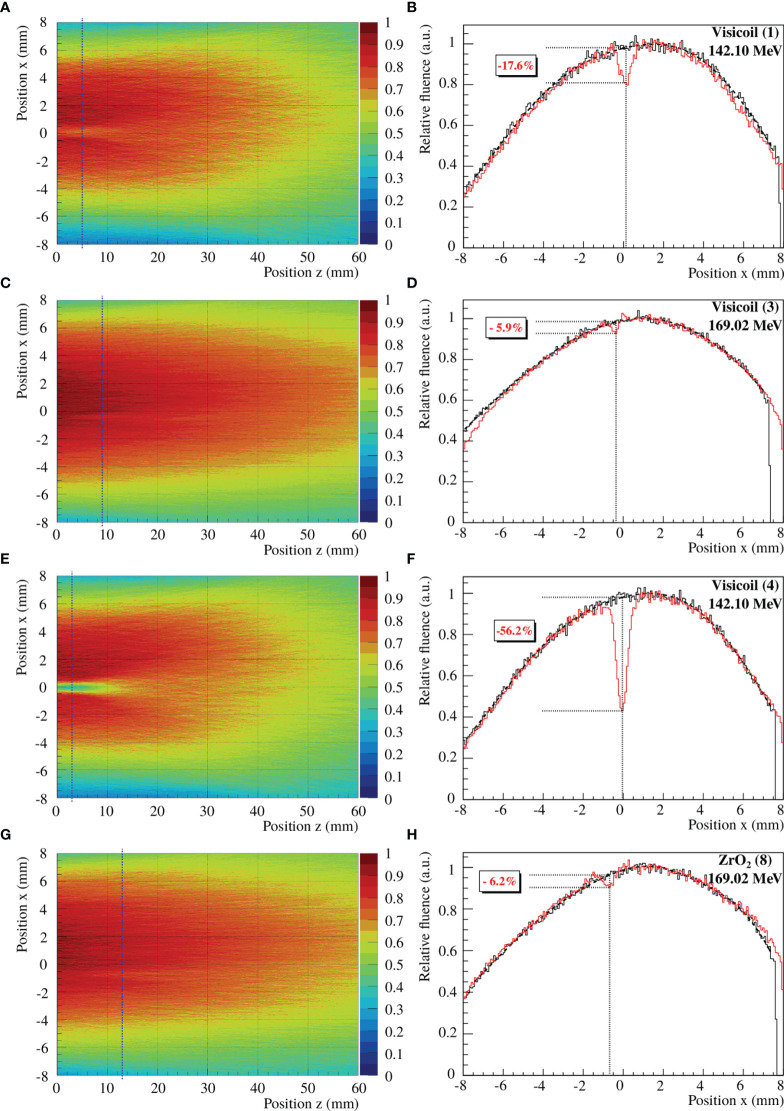
Measured fluence maps and beam profiles of 142.10 and 169.02MeV proton beams through Visicoil (1), Visicoil (3), Visicoil (4), and ZrO_2_ (8), placed at position zero along the *z*-axis and *x*-axis. **(A, C, E, G)** show the fluence maps reconstructed from all tracks. **(B, D, F, H)** show their corresponding profile at the *z* position where the perturbation is the strongest. The black vertical dash-dotted line on the fluence map represents the corresponding position along the beam axis where the fluence perturbation is maximum. In **(B, D, F, H)**, the red line shows the profile at this position with the marker, while the black line shows the profile without marker for the same *z* position. In the same panels, the vertical dotted line indicates the position in *x* of the maximum perturbation while the dotted horizontal lines quantify the strength of the perturbation.

In this study, the perturbation of proton beams was measured for 6 fiducial markers and two different energy proton beams of 142.10 and 169.02 MeV. To determine the maximum perturbation, the beam profile with marker was compared to the beam profile without marker for the same integrated window at the same position along the beam axis. In **Figure 8**, an example of fluence maps and the corresponding profiles from the maximum fluence perturbations are shown for 4 fiducial markers and different energy proton beams. The zero positions in *x* and *z* are the coordinates of the fiducial marker position. The statistical uncertainties on the maximum perturbation values were calculated as the quadratic sum of the uncertainty on the beam profiles with and without marker. The uncertainty on the *z* position, where the maximum perturbation is present, was determined as 1.5 mm, comprising the uncertainty of the sensor positioning and the uncertainty of the reconstructed track. A summary of the maximum fluence perturbation values from the fiducial markers and their corresponding position along the beam axis is listed in **Table 5**. These values were obtained from the back-projected tracks to the marker position, measured in air (see experimental setup in **Figure 2A**). The maximum perturbations and their position vary as a function of the markers and the primary beam energy. The lower the energy is, the stronger is the effect induced by the marker. Moreover, markers with high density and high atomic number create stronger and larger perturbations. For 142.10MeV protons, the perturbation is significantly higher for the fiducial markers with diameter ≥ 0.5 mm, which additionally stop the low-energy particles and induce a significant range shift.

**Table 5:** Maximum fluence perturbation values and their position along the beam axis for the different fiducial markers for 142.10 and 169.02 MeV proton beams.

Marker	142.10 MeV protons		169.02 MeV protons	
	Perturbation (%)	*z* position (mm)	Perturbation (%)	*z* position (mm)
Visicoil (1)	17.6 ± 1.3	5 ± 1.5	3.1 ± 1.4	9 ± 1.5
Visicoil (2)	52.5 ± 1.2	3 ± 1.5	14.5 ± 1.3	8 ± 1.5
Visicoil (3)	20.2 ± 1.3	5 ± 1.5	5.9 ± 1.4	9 ± 1.5
Visicoil (4)	56.2 ± 1.2	3 ± 1.5	15.4 ± 1.4	9 ± 1.5
Gold Anchor (5)	17.1 ± 1.3	2 ± 1.5	7.3 ± 1.4	7 ± 1.5
ZrO_2_ (8)	58.5 ± 1.1	3 ± 1.5	6.2 ± 1.4	13 ± 1.5

The given error on the perturbation value is the statistical uncertainty.

#### 3.2.2 2D Range Modulator

**Figure 9:**
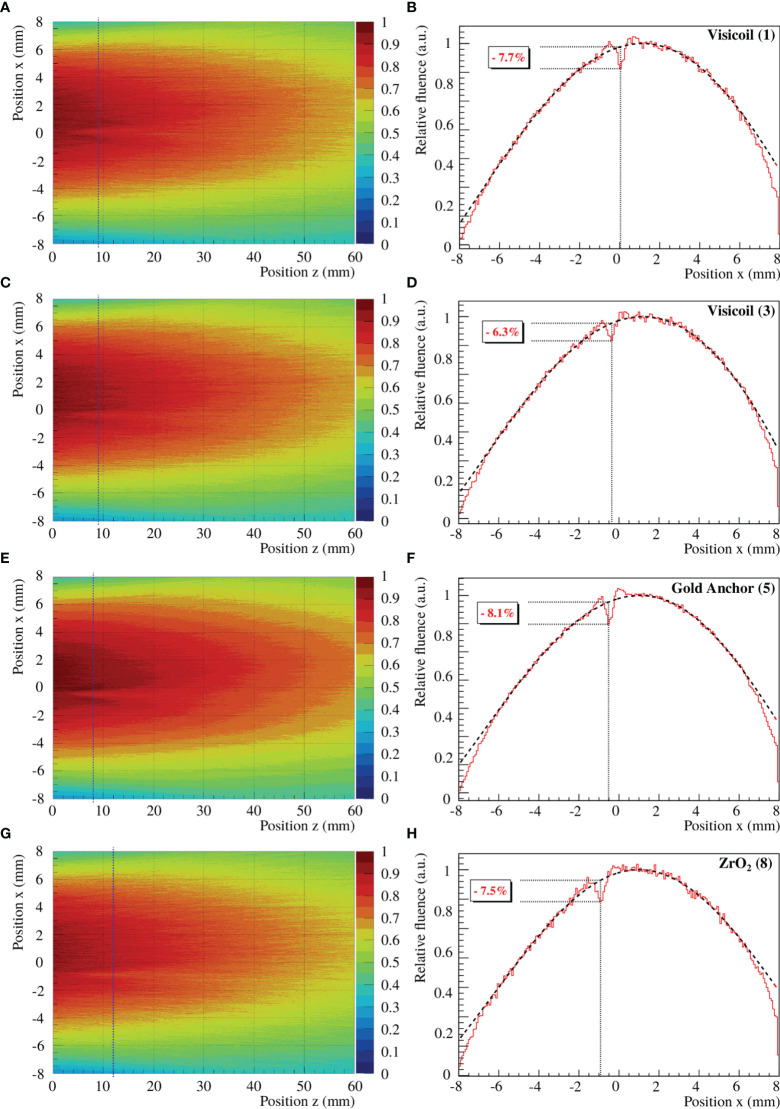
**(A, C, E, G)** show the fluence maps reconstructed from all tracks. **(B, D, F, H)** show their corresponding profile at the z position where the perturbation is the strongest. The black vertical dash-dotted line on the fluence map represents the corresponding position along the beam axis where the fluence perturbation is maximum. In **(B, D, F, H)**, the red line shows the profile at this position with the marker, while the black line shows the profile without marker for the same z position. In the same panels, the vertical dotted line indicates the position in x of the maximum perturbation while the dotted horizontal lines quantify the strength of the perturbation.

In this study, the perturbation of proton beams was measured for 4 fiducial markers, and the fluence maps were reconstructed for a 170.05MeV proton beam modulated by a 2D RM in order to obtain a more realistic case (see **Figure 2B** for the position of the marker). To determine the maximum fluence perturbation, the beam profile with the marker was computed for a certain integrated window. As explained in *Section 2.3.4*, the reference measurement was considered as the fit of the beam profile without taking into account the perturbation. In **Figure 9**, the fluence maps and the corresponding profiles of the maximum fluence perturbations are shown for the studied fiducial markers. The uncertainties were computed as the ones obtained for the single-energy beams. A summary of the maximum perturbation values of the fiducial markers and their corresponding position along the beam axis are listed in **Table 6**. As for the single-energy beams, these values were obtained from the back-projected tracks to the marker position, measured in air (see experimental setup in **Figure 2A**).

**Table 6:** Maximum fluence perturbation values and their position along the beam axis for the different fiducial markers for the 170.05MeV proton beam modulated by a 2D RM.

Marker	Perturbation (%)	*z* position (mm)
Visicoil (1)	7.7 ± 1.4	9 ± 1.5
Visicoil (3)	6.3 ± 1.4	9 ± 1.5
Gold Anchor (5)	8.1 ± 1.3	8 ± 1.5
ZrO_2_ (8)	7.5 ± 1.3	12 ± 1.5

The given error on the perturbation value is the statistical uncertainty.

### 3.3 Monte Carlo Simulations

#### 3.3.1 Initial Beam Parameter Tuning

Monte Carlo simulations were performed with the FLUKA code (23–25), as described in Section 2.4. The initial beam optics were tuned to reproduce the experimental data as explained in Section 2.4.2. In **Table 7**, the initial beam width and beam divergence, extracted from Scattman (1, 34) and introduced in the FLUKA simulations, are listed for 142 and 170 MeV. The initial beam optics for 169 and 170 MeV were found to be the same.

**Table 7:** Initial ion beam optics (FWHM and divergence at the vacuum exit window) used for the Monte Carlo simulations for 142 and 170 proton beams.

Energy (MeV)	FWHM (mm)	Divergence (mrad)
142	3.5	7.0
170	3.5	7.5

**Figure 10:**
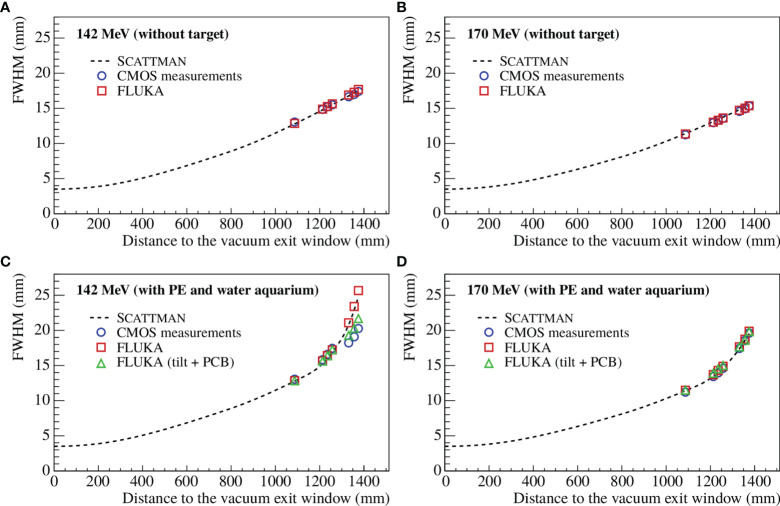
Beam width as a function of the distance to the vacuum exit window for 142 MeV **(A, C)** and 170 MeV **(B, D)** with the initial beam optics from **Table 7**. The beam width was computed for Scattman, the CMOS measurements, and the FLUKA simulation without target **(A, B)** and with the PE block and the water aquarium **(C, D)**. The Monte Carlo simulations were performed with and without including the initial beam tilt and the PCB of the sensors.

In **Figure 10**, the beam width as a function of the distance to the vacuum exit window was computed for 142 and 170 MeV with the initial ion beam optics listed in **Table 7**. The results are shown for the Scattman code, as well as for the CMOS measurements and the FLUKA simulations. The Monte Carlo simulations were performed with and without including the initial beam shift (and tilt) and the PCB of the sensors (as explained in Section 2.4.1).

In all cases, the beam width of the FLUKA simulations and the CMOS measurements are in good agreement. The good agreement between Scattman and the measurements were expected since the ion optical parameters in the Scattman model were adapted from the CMOS measurements. In **Figure 10C**, deviations can be observed between the FLUKA simulations and the CMOS measurements. However, these deviations are significantly reduced when the simulation takes into account the PCB layer and the initial beam tilt. For 142 MeV with the PE block and the water aquarium, the proton beam has an energy of around 8 MeV in the last sensor, which is equivalent to around 2mm range in water. Therefore, the PCB of the first set of sensors plays a role in the energy loss, and this effect becomes significant only for low-energy beams.

#### 3.3.2 Fluence Perturbation Comparison With Marker

**Figure 11:**
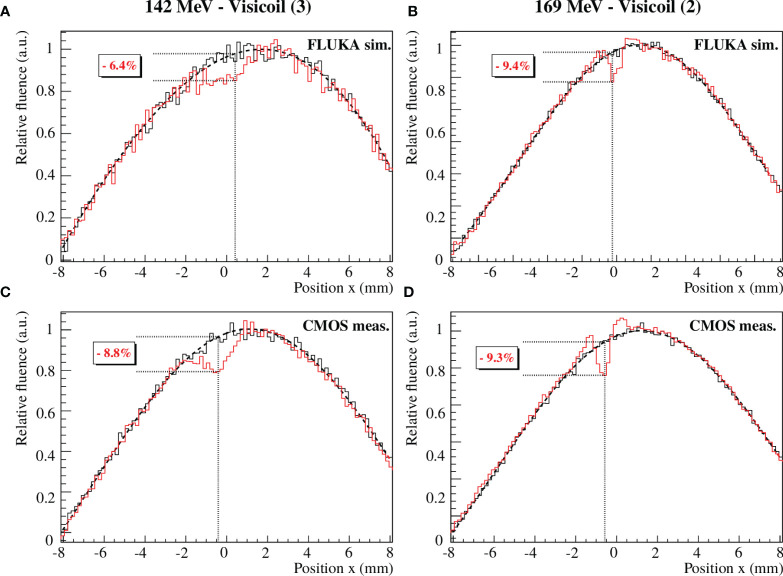
Beam profile comparison between the FLUKA simulations **(A, B)** and the CMOS measurements **(C, D)** for 142MeV protons passing through the Visicoil (3) **(A, C)** and 169MeV protons passing through the Visicoil (2) **(B, D)**. The red and black lines show the beam profiles with and without marker, respectively.

The FLUKA simulations with fiducial markers were compared to the CMOS measurements following the simulated setup described in *Section 2.4.1*. The beam profiles were obtained with the Monte Carlo simulations and the CMOS measurements as explained in *Section 2.4.2*. In **Figure 11**, the beam profiles at sensor 5 position (see **Figure 2A**) are shown for the Visicoil (3) for 142MeV protons and for the Visicoil (2) for 169MeV protons.

In **Tables 8**, **9**, the fluence perturbation values are compared for the FLUKA simulations and the CMOS measurements for 142 and 169MeV protons, respectively. The fluence perturbations were computed for several positions (sensor 5, sensor 6, and sensor 7) to compare its propagation along the beam axis.

**Table 8:** Comparison of the fluence perturbation values at several positions (sensor 5, sensor 6, and sensor 7) between the FLUKA simulations (Sim.) and the CMOS measurements (Meas.) for the different fiducial markers for the 142MeV proton beam.

Marker	Perturbation on S5 (%)		Perturbation on S6 (%)		Perturbation on S7 (%)	
	Sim.	Meas.	Sim.	Meas.	Sim.	Meas.
Visicoil (1)	8.2 ± 1.4	9.0 ± 1.4	3.4 ± 1.4	4.2 ± 1.4	1.8 ± 1.4	3.7 ± 1.4
Visicoil (2)	15.8 ± 1.3	19.1 ± 1.3	8.0 ± 1.4	7.2 ± 1.4	4.1 ± 1.4	4.7 ± 1.4
Visicoil (3)	6.4 ± 1.4	8.8 ± 1.4	3.4 ± 1.4	5.0 ± 1.4	3.1 ± 1.4	3.1 ± 1.4
Visicoil (4)	17.2 ± 1.3	21.4 ± 1.3	6.8 ± 1.4	8.3 ± 1.4	3.8 ± 1.4	3.9 ± 1.4
Gold Anchor (5)	9.4 ± 1.4	11.3 ± 1.3	3.8 ± 1.4	5.2 ± 1.4	2.4 ± 1.4	3.0 ± 1.4
ZrO_2_ (8)	14.0 ± 1.4	24.9 ± 1.3	6.4 ± 1.4	9.7 ± 1.4	3.8 ± 1.4	2.4 ± 1.4

The given error on the perturbation value is the statistical uncertainty.

**Table 9:** Comparison of the fluence perturbation values at several positions (sensor 5, sensor 6, and sensor 7) between the FLUKA simulations (Sim.) and the CMOS measurements (Meas.) for the different fiducial markers for the 169MeV proton beam.

Marker	Perturbation on S5 (%)		Perturbation on S6 (%)		Perturbation on S7 (%)	
	Sim.	Meas.	Sim.	Meas.	Sim.	Meas.
Visicoil (1)	4.6 ± 1.4	3.7 ± 1.4	2.0 ± 1.4	2.5 ± 1.4	2.2 ± 1.4	1.6 ± 1.4
Visicoil (2)	9.4 ± 1.4	9.3 ± 1.4	4.6 ± 1.4	4.0 ± 1.4	3.6 ± 1.4	3.3 ± 1.4
Visicoil (3)	5.2 ± 1.4	4.6 ± 1.4	2.5 ± 1.4	2.8 ± 1.4	2.1 ± 1.4	2.5 ± 1.4
Visicoil (4)	12.3 ± 1.4	12.1 ± 1.4	5.4 ± 1.4	6.9 ± 1.4	3.9 ± 1.4	4.6 ± 1.4
Gold Anchor (5)	5.2 ± 1.4	4.2 ± 1.4	2.1 ± 1.4	2.4 ± 1.4	1.5 ± 1.4	1.6 ± 1.4
ZrO_2_ (8)	6.1 ± 1.4	5.3 ± 1.4	3.1 ± 1.4	3.2 ± 1.4	2.9 ± 1.4	2.5 ± 1.4

The given error on the perturbation value is the statistical uncertainty.

The results show a good agreement between the Monte Carlo simulations and the CMOS measurements within the uncertainties. For 142MeV protons, the deviations are bigger than for 169 MeV. This was expected since the primary protons of 142 MeV have an energy of around 10 MeV behind the water aquarium, compared to around 80 MeV in the case of the 169MeV primary proton beam. This means that small variations of the layer thicknesses of the simulated geometry, in the case of 142MeV protons, lead to larger fluctuations of the energy losses compared to the 169MeV proton beam. Therefore, small geometrical uncertainties in the simulated setup have a stronger impact on the perturbation values for low-energy beams. However, the results from both the simulations and the experiments are in good agreement, showing the same tendencies. The Monte Carlo simulations could reproduce correctly the perturbation and its propagation along the beam axis.

#### 3.3.3 Dose Perturbation: FLUKA Simulations

**Figure 12:**
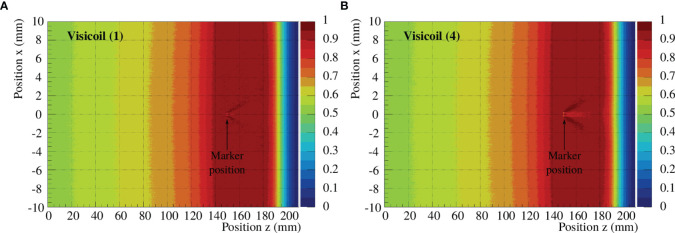
Dose maps for an SOBP created with protons in water, covering 5 cm in depth. The pristine Bragg curves were generated with the Monte Carlo code FLUKA and superimposed to obtain the SOBP. The dose maps are shown for the Visicoil (1) **(A)** and the Visicoil (3) **(B)**, placed at 15 cm depth.

Compared to our previous work (20), where only fluence measurements were performed, dose distributions were also simulated in the present work. The dose distributions were computed following the description in **Section 2.4.3**, where a 5cm SOBP was created with protons and the fiducial markers were placed at the beginning of the SOBP at the 15cm depth. The simulated depth–dose profiles showed a very similar distribution to the measured ones from other experiments (32). In **Figure 12**, the dose maps are shown for the gold Visicoil (∅ = 0.35 mm) and the platinum Visicoil (∅ = 0.5 mm).

**Figure 13:**
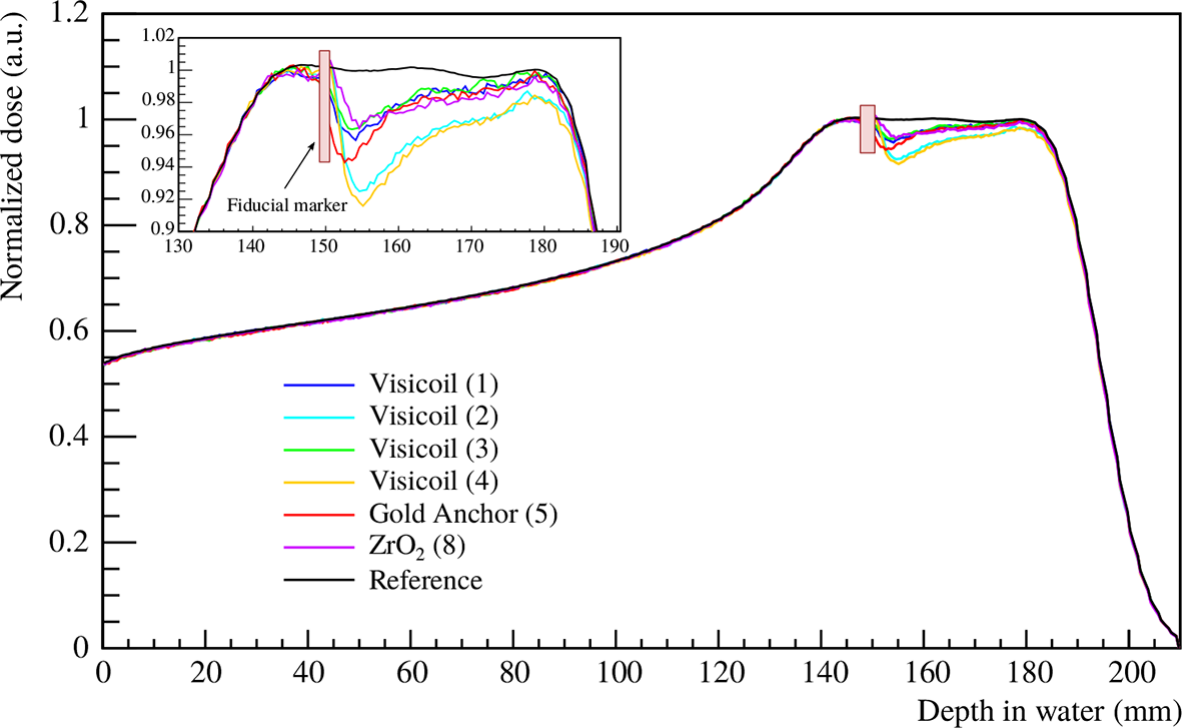
SOBP created with protons in water, covering 5 cm in depth. The depth–dose profiles were extracted from the central line of the dose maps. The black line shows the reference measurement without marker, while the colored lines show the SOBP with a fiducial marker placed at 15cm depth. The FLUKA simulations were performed for 6 fiducial markers (**Table 1**).

In **Figure 13**, the depth–dose profiles in the central line obtained from the dose maps are presented for all the studied markers. A reference simulation without marker is also shown in the same figure. The maximum perturbations and their position behind the fiducial marker along the beam axis are listed in **Table 10** for 6 fiducial markers. The two Visicoil markers of 0.5mm diameter (2) and (4), which are the heaviest markers used in this study, show the strongest perturbation of around 8%. The Gold Anchor (5) shows a maximum cold spot of around 6%, while the other lighter markers (1), (3), and (8) produce a perturbation of around 3–4%. The dose perturbations presented in this section show the same tendency as for the fluence measurements presented in *Section 3.2.2*.

**Table 10:** Maximum cold spot values and their position behind the fiducial marker along the beam axis for 6 different markers (**Table 1**), computed with FLUKA simulations.

Marker	Cold spot (%)	*z* position (mm)
Visicoil (1)	4.3 ± 1.4	4.1 ± 0.5
Visicoil (2)	7.5 ± 1.4	4.6 ± 0.5
Visicoil (3)	3.6 ± 1.4	3.6 ± 0.5
Visicoil (4)	8.4 ± 1.4	5.1 ± 0.5
Gold Anchor (5)	5.7 ± 1.4	2.6 ± 0.5
ZrO_2_ (8)	3.4 ± 1.4	4.6 ± 0.5

The given error on the cold spot value is the statistical uncertainty.

## 4 Discussion

In this work, a comprehensive study was performed for fiducial markers used for image guidance in proton beam therapy. Three main criteria for these markers, which are streak artifacts on treatment planning CT, visibility on daily imaging methods, and dose perturbations during particle therapy, were evaluated. Fiducial markers composed of different materials (gold, platinum, and ZrO_2_) and with different geometries were studied. The markers of high-density materials (such as gold and platinum) had a diameter ≤0.5 mm, which is recommended for particle therapy (8, 20).

The streak artifacts on treatment planning CT, reconstructed with and without iMAR correction, were quantified for 7 fiducial markers. The maximum and minimum HU were computed as a function of the distance to the fiducial markers. The gold and platinum Visicoil of 0.5mm diameter and the two Gold Anchor markers, which have a mass bigger than 6 mg, were the ones inducing the strongest streak artifacts. The distance at which the streak artifacts were lower than 3% of the background level was found to be bigger than 10 mm for these markers, and 7 mm for the gold and platinum Visicoil of 0.35mm diameter and the ZrO_2_ markers, which were lighter than 6 mg. For the case of treatment planning CT with iMAR correction, the streak artifacts are significantly reduced. The distance at which they are lower than 3% of the background level is reduced by a factor 2 compared to the conventional treatment planning CT in all cases. However, the iMAR correction would need to be further investigated to be applied for clinical treatment planning. It is important to note that the markers are smaller than a CT voxel; however, they appear in up to 4 neighboring voxels. Before treatment planning, the voxels need to be overwritten by a realistic value, considering the partial volumes and the stopping power of the marker material relative to water. This could lead to inaccurate range predictions around the marker.

The visibility of fiducial markers on X-ray projections was measured in 4 different scenarios with and without collimation, as well as with and without bone slabs in front of the markers. As expected, the contrast of the fiducial markers with bone slabs is significantly reduced. In a realistic case, with bone slabs and collimation, the heavier the marker is and higher is its density, the stronger the contrast is. However, all markers were visible in all cases.

The fluence perturbation from different fiducial markers was measured for proton beams with an advanced technique by using high resolution CMOS pixel sensors. With this method, 3D fluence distributions could be computed after reconstructing the trajectory of each particle. The measurements were performed with two single-energy beams and with a modulated beam using a 2D range modulator, producing an SOBP of 5cm length. The maximum fluence perturbation, created behind the fiducial marker and its position along the beam axis, was quantified, using the 2D fluence map. As in our previous study (20), the perturbations creating a small overdosage were not reported since it is less critical than a local underdosage that could cause a recurrence of the tumor. The created fluence perturbations can be caused by edge scattering but also due to particles stopping in the marker when the particle beam is at the end of its range. Both phenomena were observed in this study for the single-beam energy experiments, using 142.10 and 169.02MeV protons. For fiducial markers of diameter smaller than 0.5 mm, the perturbations were found to be lower than 8%, while the two Visicoil markers of 0.5 mm showed a maximum perturbation of around 15% for 169.02MeV protons. In the case of the lower energy proton beam, the maximum fluence perturbation was found to be around 50% for the markers thicker than 0.5 mm. These strong perturbations were due to high-energy losses in the fiducial markers, which fully stopped the low-energy proton beam. The perturbations induced by the Visicoil markers of 0.35mm diameter, the Gold Anchor, and the ZrO_2_ marker were also measured for a modulated beam. These measurements provided a more realistic situation of a mixed beam, where the markers were positioned at the entrance of a 5cm SOBP. The created fluence perturbations were found to be between 6% and 8% in all cases, which is closer to the perturbations created for 169.02MeV protons. This was expected since the marker was positioned in the plateau region of the pristine Bragg peak with the higher weight in the SOBP. The maximum perturbation depends on the position of the fiducial marker. The closer the marker is to the end of the SOBP, the bigger the perturbation is due to the stronger scattering and higher energy losses of the protons with the higher weighted pristine Bragg peak (36).

In addition, Monte Carlo simulations were performed with FLUKA (25) to compute a 5cm length SOBP, from 14 to 19 cm, created with protons in a water target. Dose perturbations induced by the fiducial marker were computed, and the created cold spots were quantified. The simulations, with and without fiducial marker, were first validated against the experimental measurements performed with the CMOS sensors. The FLUKA simulations and CMOS measurements were in good agreement. The perturbation of the different fiducial markers was computed for a marker placed at 15cm depth at the beginning of the SOBP. The maximum cold spot was found to be around 8% for the heaviest markers and around 4% for the smallest ones.

In this work, the perturbations were measured and simulated for a single field. However, in a realistic case, a patient would be treated with multiple fields, partly compensating the perturbation from one field to another (37). Moreover, daily alignment, which is generally performed with the bony structure of the patient, can be performed with an accuracy of only 0.5 cm. This would also blur out the effect during the particle therapy treatment between different fractions. However, the perturbations remain the same in the tissue for every fraction.

## 5 Conclusions

In this work, streak artifacts on treatment planning CT, visibility on daily imaging, and dose perturbations during proton therapy were studied for different fiducial markers that are used for image guidance in radiotherapy. The markers of different geometries were composed of different materials such as gold, platinum, and ZrO_2_. The streak artifacts on a treatment planning CT were found to be the lowest for fiducial markers with a mass lower than 6 mg. However, the treatment planning CT reconstructed with iMAR correction showed a significant reduction of the streak artifacts for all markers. Visibility on X-ray projections was also evaluated, and a better contrast for heavier markers was found. However, all markers were visible in all studied scenarios. Imaging with in-room mobile CT was also performed showing a clear visibility from all markers. An advanced measurement method was used to quantify fluence perturbations due to fiducial markers in proton therapy, using high resolution CMOS pixel sensors. Based on the fluence maps, the measurements showed that the perturbations due to fiducial markers are reduced for small and low-density markers of mass lower than 6 mg. Monte Carlo simulations were performed with a comparable setup as the one used during the experiments. The simulations were validated against the experiments with and without fiducial marker, showing good agreement with the experimental data after adjusting properly the ion optical parameters (beam width and divergence). Dose maps were simulated for an SOBP in water created with protons, where the fiducial marker was positioned at the beginning of the SOBP. From this work, fiducial markers with a low mass, such as the gold and platinum Visicoil of 0.35mm diameter as well as ZrO_2_ (carbon-coated), can be recommended for image guidance in proton therapy since they provide a good trade-off between visibility versus dose perturbation. In principle, even smaller markers could be used as long as they are still visible on the daily imaging method.

## Data Availability Statement

The original contributions presented in the study are included in the article/supplementary material. Further inquiries can be directed to the corresponding author.

## Author Contributions

Conceptualization and methodology: C-AR, FH, CS, and UW. Investigation: C-AR, FH, and CS. Resources: OJ, SE, KH, and SB. Project administration, OJ, SE, KH, SB, MD, and UW. Original draft of the manuscript: C-AR. All authors contributed to the article and approved the submitted version.

## Conflict of Interest

The authors declare that the research was conducted in the absence of any commercial or financial relationships that could be construed as a potential conflict of interest.

## Publisher’s Note

All claims expressed in this article are solely those of the authors and do not necessarily represent those of their affiliated organizations, or those of the publisher, the editors and the reviewers. Any product that may be evaluated in this article, or claim that may be made by its manufacturer, is not guaranteed or endorsed by the publisher.
